# Taking the Case

**Published:** 1888-01

**Authors:** Robert Farley

**Affiliations:** Phœnixville, Pa.


### TAKING THE CASE.

To the Hahnemannian Association of Pennsylvania:—
This subject is of vital importance to every homœopathic physician and yet how terribly is it neglected by the vast majority of them, because of their carelessness and laziness; or through their ignorance of its positive necessity in order to make rapid, gentle, and permanent cures. We, as homœopathic physicians, believe the *totality* of the symptoms presented by the sufferer to be the infallible guide in choosing the curative remedy, and yet how few of us *get* the totality of the symptons to guide us in our choice of the remedy. We believe the remedy which will cure the case quickly, gently, and permanently is the one whose symptoms most nearly resemble the symptoms presented by our patient.

Now it is very evident to us all, that we cannot choose the most similar remedy in a given case if we do not have *all* the symptoms of the case for comparison, or, in other words, we cannot fit the foot with a shoe if we are in ignorance as to the size of the foot. Hahnemann advised his followers to take a written record of each case, but that entails too much labor and is too slow and prosy for this "quick-stepping" generation. We prefer to make "snap shots," shoot often—and usually miss the bird.

It is true, that all physicians have not the faculty of taking a clear and perfect picture of the case in hand, but we could nearly *all* of us do much better than we do, if we would appreciate the responsibility and grandeur of our calling, that of saving human life and alleviating suffering, and would study the writings of our master, Hahnemann, more earnestly, with the desire to thoroughly comprehend our art. Hahnemann has said: "When we have to do with an art whose end is the saving of human life, any neglect to make ourselves thoroughly masters of it becomes a crime."

We have said that all physicians have not the ability to properly take a case for the homœopathic prescription. Now if this be true, what are the requisites necessary in the physician for the proper taking of the case? He must possess the ability to read human nature in its many phases, for his patients will be of all sizes, shapes, and kinds and of both sexes; keen powers of observation, educated by daily and hourly practice; fidelity in noting the case, unbiased judgment, and with all, wonderful tact in order to deal with peculiarities successfully and not cause offense. He must command respect and confidence and cause the patient to feel that he has his (the patient's) greatest good at heart, and that his examination of the case is only intended to enable him to apply the means that will restore him to health.

We have tried to picture the ideal examiner; now, how shall the examination be conducted?

What will be said relates principally to chronic cases, for they are vastly more intricate, mixed up, and harder to faithfully picture. In acute cases the important, chief, and characteristic symptoms are prominent and usually easily comprehended, while in chronic cases, the characteristic symptoms, the ones that will be our chief guide in the selection of the homœopathic remedy, are often far from prominent; and, owing to the long-suffering of the patient, they may not be associated in his mind with any abnormal state, and may have to be brought out by questioning and cross-questioning.

In fact, we often need the skill of a lawyer in eliciting and weighing evidence. Some of our clients will greatly exaggerate their suffering and symptoms in order, as they think, to excite our sympathy and cause us to make strenuous efforts for their relief. Here we will have to do more or less subtracting to get a true picture of the case. Some others, again, will try to state the case to make it correspond to their preconceived ideas of what they believe is the disease from which they are suffering, or to make it conform to what they may think your idea of it is. Others will warp the case from dread of or shame for the truth; and some patients, for fear of appearing to be of a complaining disposition, will make light of their suffering and will in this way unintentionally baffle us if we are not extremely wide-awake to the situation. But the most aggravating of all, is the patient so astute as to be unable to describe his symptoms; he cannot deal in figurative speech at all; or he is so unobserving of himself that he cannot tell his physician whether he has an evacuation from his bowels each day or not. These are some of the difficulties to be overcome in taking the case.

When our patient begins the narration of his symptoms we must not interrupt him in any way, unless he wanders too far from the subject, but let him finish it in his own way, lest we interrupt the thread of his thought and he be not able to take it up again. Neither will it do to seem to comprehend his meaning too readily, or he will be less explicit and accurate in his recital, thinking we know it all without being told. During his recital of his symptoms we should be occupied in taking careful notes of the symptoms as he mentions them, leaving plenty of space under the different heads, to be filled in later, after he has finished his recital. If the patient talks too rapidly we should caution him to speak more slowly, so that we may not miss anything in our notes. We must not consider any symptom too trifling for our notice, for who of us knows how important that, apparently trifling, symptom may be later on in the case; it may be the symptom that decides us in our choice of the simillimum to the case. But while we must note all symptoms, we must also be careful to exclude any spurious symptoms that may be given us by our patients.

Now after our patient has finished his recital there will be many important omissions, and if we have, as we should, left vacant space after each group or series of symptoms, we now, by questioning and cross-questioning, taking care to avoid leading or direct questions, will be able to complete our picture of the case.

A symptom is any deviation from the normal in function or in tissue, however discoverable, whether by sight, palpation, auscultation or by olfaction, whether subjective or objective; therefore we must use every means at our command to enable us to get the totality of the symptoms of each case. Without this completeness of examination many symptoms would mislead us, especially in our diagnosis and prognosis.

After this thorough taking of the case we have then to decide what symptoms are the characteristic and peculiar ones of each individual case. Our knowledge of materia medica will greatly aid us in the examination of the case. The recital of the case will call to our minds more or less perfect pictures of perhaps a number of remedies, and we will instinctively begin a comparison of these remedies with the case in hand, and thus bring out that which excludes some of the remedies and increase the similarity of others. By this running comparison we often bring out symptoms of importance that had been omitted and fill up and round off the features, and thus get a perfect picture of the case. Often by this time we have decided what drug in our materia medica presents a similar picture, if not, we must turn to it with the picture of the case and compare it with drug-pictures until we find its image reflected by a drug and our search is over.

In children and adults who are, from any cause, unable to observe and detail their own symptoms, we have recourse to their parents or friends who are with them, to state what they have observed and what complaints they have heard from the patient, thus helping us to get the case and doing what, under other circumstances, the patient would have done.

Now after all our care and painstaking the picture may be a very imperfect one of the disease, due to the drugging the patient may have recently undergone. We may find it necessary to antidote the drug action and wait, to simply wait, or, to prescribe for the complex of drug symptoms and original disease symptoms and later, when our remedy has done all it can do, re-examine the case for the homœopathic remedy.

Another thing absolutely essential to the taking of a perfect picture, of the chronic case especially, is the history of the case. The different features of the case may make themselves manifest through a long period of time, months or even years; for example, one set of symptoms in the relaxing weather of spring or summer, another during the vigorous weather of winter. Therefore, in order to get a picture of the chronic case we must extend our observation of a long period of time. Dunham has reported an old case of cephalalgia, cured by him, with Aloes, the prescription being based on a diarrhœa from which the patient suffered every summer, the prescription being made during the winter, which was the time of the year the cephalalgia made itself manifest.

It occasionally happens that the disease of the patient is wholly due to errors in diet or in his habits in life, and a correction of these errors be all that is necessary for a return to health. The scientific physician will always endeavor to remove the cause of disease, wherever practicable, before he applies his homœopathic remedy for the case. This will indicate that a careful inquiry into the diet and habits of our patient's life should always constitute an important part of our examination of the case.

We know that some of the curable chronic diseases require months and even years for their removal; therefore, unless we take such cases in writing it is impossible to treat them with any satisfaction to a homœopathic physician. After the administration of the indicated remedy, and our patient calls again in a week, two weeks or a longer time, we can then refer to our record of the case and go carefully over it with our patient, ascertaining whether any changes or new symptoms have occurred, noting them and crossing off any that have disappeared for a sufficient length of time to warrant us in so doing; we then have a new picture and are ready to determine whether the remedy is still acting favorably, and we are to let it continue to do so, undisturbed, or, repeat the remedy, or whether this new picture demands a re-selection of the homœopathic remedy.

The lack of attention paid to the subject of this paper was very forcibly impressed upon my mind some months ago, when I attended a meeting of "The Homœopathic Medical Council," where a chronic case of long standing was presented for consultation, and I, very naturally, asked the physician who had been treating the case, he being an earnest disciple of Hahnemann, for his record of the case. His reply, "I have never written it up," surprised me, to say the least. Since that time the lack of attention paid to this important subject has been called to my mind several times, and when your worthy President requested me to present a paper to the "Association," I concluded to use the opportunity to present the subject for your consideration.

Our object in coming together in these meetings is for mutual benefit and the advancement of our beloved profession, the practice of pure Homœopathy; therefore, if in spite of the rambling and disjointed character of this paper, we have succeeded in impressing the very great importance of properly *taking the case* upon the mind of any homœopathic physician, we feel that something has been done for the advancement of pure Homœopathy, and are content.

Fraternally,

PHŒNIXVILLE, PA. ROBT. FARLEY, M. D.

